# Acute use of teriparatide and palopegteriparatide in the management of hungry bone syndrome following parathyroidectomy

**DOI:** 10.1210/jcemcr/luag089

**Published:** 2026-04-28

**Authors:** Sarah Jacob, Jennifer Rowell, Nicole Sagalla

**Affiliations:** Division of Endocrinology, Metabolism and Nutrition, Duke University Hospital, Durham, NC 27710, USA; Division of Endocrinology, Metabolism and Nutrition, Duke University Hospital, Durham, NC 27710, USA; Division of Endocrinology, Metabolism and Nutrition, Duke University Hospital, Durham, NC 27710, USA

**Keywords:** postoperative hypocalcemia, hungry bone syndrome, teriparatide, palopegteriparatide

## Abstract

Hypocalcemia remains a major risk of head and neck surgery, especially after parathyroidectomy for treatment of hyperparathyroidism. In this case, we present a patient with hyperparathyroidism who developed hungry bone syndrome postoperatively, requiring more than 15 grams of calcium supplementation per day but who only sustained normal calcium levels with the addition of teriparatide followed by palopegteriparatide. This is 1 of only a few case reports in the literature describing acute use of teriparatide or palopegteriparatide in the management of severe hungry bone syndrome.

## Introduction

Postoperative hypocalcemia can be a complication after parathyroidectomy or thyroidectomy. Most cases of hypocalcemia are temporary and often due to transient stunning of the parathyroids related to surgical manipulation or due to suppression of remaining glands in the case of hyperparathyroidism [1-3]. Patients undergoing parathyroidectomy for hyperparathyroidism may develop hypocalcemia due to hungry bone syndrome (HBS). The pathophysiology of HBS is thought to be due to sudden shifts toward osteoblastic activity after surgical cure of hyperparathyroidism or hyperthyroidism, and the condition is defined by hypocalcemia with normal parathyroid hormone (PTH) that lasts for more than 4 days postoperatively [4, 5]. Risk factors for HBS include larger parathyroid gland, higher preoperative PTH and alkaline phosphatase, and bone disease on imaging [4]. Management is similar to postoperative hypocalcemia from other causes, but in practice, HBS is often more difficult to control.

PTH analogs have been used to successfully manage postthyroidectomy hypocalcemia refractory to standard therapies [6]; however, limited evidence exists in the literature for the use of PTH analogs for acute hypocalcemia and HBS. In this case report, we present a case of HBS following parathyroidectomy for hyperparathyroidism in a patient with chronic kidney disease (CKD) stage IV, who failed standard therapy and had subsequent improvement in calcium with the addition of teriparatide and palopegteriparatide.

## Case presentation

A 28-year-old male with a past medical history of sickle cell disease, hypertension, CKD stage IV, and hyperparathyroidism underwent parathyroidectomy for hypercalcemia management.

Hypercalcemia was initially noted 5 years prior to surgery (Table 1). PTH was first measured 8 months prior to surgery and was elevated along with serum calcium and creatinine. During the 5-year time period between the first incidence of hypercalcemia and surgery, serum calcium remained elevated between 10.4 and 11.9 mg/dL [International System of Units (SI): 2.59-2.97 mmol/L] (reference range, 8.7-10.2 mg/dL [SI: 2.17-2.54 mmol/L]), albumin was normal between 3.6 and 4.1 g/dL (SI: 36-41 g/L) (reference range, 3.5-4.8 g/dL [SI: 35-48 g/L]), vitamin D was deficient at <20 ng/mL (SI <50 nmol/L) (reference range, 30-100 ng/mL [SI: 74.9-249.6 nmol/L]), alkaline phosphatase was elevated between 313 and 413 U/L (SI: 5.2-6.9 μkat/L) (reference range, 24-110 U/L [SI: 0.4-1.83 μkat/L]), and creatinine and glomerular filtration rate had progressed to CKD IV (Table 1). Progression of CKD was thought to be due to hypertension and sickle cell disease.

**Table 1:** Preoperative lab trends

	Serum calcium, mg/dL (SI)	Albumin, g/dL (SI)	Glomerular filtration rate (mL/min/1.73 m^2^)	25-hydroxyvitamin D, ng/mL (SI)	PTH, pg/mL (SI)	Alkaline phosphatase, U/L (SI)	Serum phosphate, mg/dL (SI)
Reference range (SI)	8.7-10.2 mg/dL (SI: 2.17-2.54 mmol/L)	3.5-4.8 g/dL (SI: 35-48 g/L)	>90 mL/min/1.73 m^2^	30-100 ng/mL (SI: 74.9-249.6 nmol/L)	14-72 pg/mL (SI: 1.5-7.6 pmol/L)	24-110 U/L (SI: 0.4-1.83 μkat/L)	2.3-4.5 mg/dL (SI: 0.74-1.5 mmol/L)
5 years prior to surgery	**11 **mg/dL (**2.7 **mmol/L)	4.6 g/dL (46 g/L)	>60 mL/min/1.73 m^2^	NA	NA	NA	NA
8 months prior to surgery	**11.2 **mg/dL (**2.8 **mmol/L)	3.9 g/dL (39 g/L)	**25 **mL/min/1.73 m^2^	**12 **ng/mL (**30 **nmol/L)	**1532 **pg/mL (**162.5 **pmol/L)	**313 **U/L (**5.2 **μkat/L)	3.3 mg/dL (1.1 mmol/L)
6 months prior to surgery	**11.8 **mg/dL (**2.9 **mmol/L)	4.0 g/dL (40 g/L)	**21 **mL/min/1.73 m^2^	NA	**1948**pg/mL (**206.6 **pmol/L)	NA	4.1 mg/dL (1.3 mmol/L)
3 months prior to surgery	**12.0 **mg/dL (**3 **mmol/L)	3.8 g/dL (38 g/L)	**16 **mL/min/1.73 m^2^	NA	**2380 **pg/mL (**252.4 **pmol/L)	**383 **U/L (**6.4 **μkat/L)	4.4 mg/dL (1.4 mmol/L)
1 month prior to surgery	**11.5 **mg/dL (**2.9 **mmol/L)	4.0 g/dL (40 g/L)	**15 **mL/min/1.73 m^2^	**10 **ng/mL (**25 **nmol/L)	**1913**pg/mL (**202.9 **pmol/L)	**413 **U/L (**6.9 **μkat/L)	3.3 mg/dL (1.1 mmol/L)
On admission for surgery	**11.4 **mg/dL (**2.8 **mmol/L)	3.4 g/dL (34 g/L)	**15 **mL/min/1.73 m^2^	NA	NA	**408 **U/L (**6.8 **μkat/L)	NA
Morning of surgery	**10.9 **mg/dL (**2.7 **mmol/L)	NA	**15 **mL/min/1.73 m^2^	NA	NA	NA	NA

Bolded typeface indicates laboratory values outside the reference range.

Abbreviations: NA, not available; SI, International System of Units.

An initial consultation with endocrinology and endocrine surgery occurred 2 months prior to surgery. At that time, the patient denied polyuria, fatigue, constipation, brain fog, or confusion but reported worsening throbbing bone and muscle pain. He had no history of kidney stones, fractures, head and neck surgery, radiation, or family history of parathyroid disease. He had been trialed on calcitriol and vitamin D for management of hyperparathyroidism with his nephrologist, but both agents were stopped in the setting of hypercalcemia. Cinacalcet had been ordered but not started due to a lack of insurance coverage.

**Figure 1:**
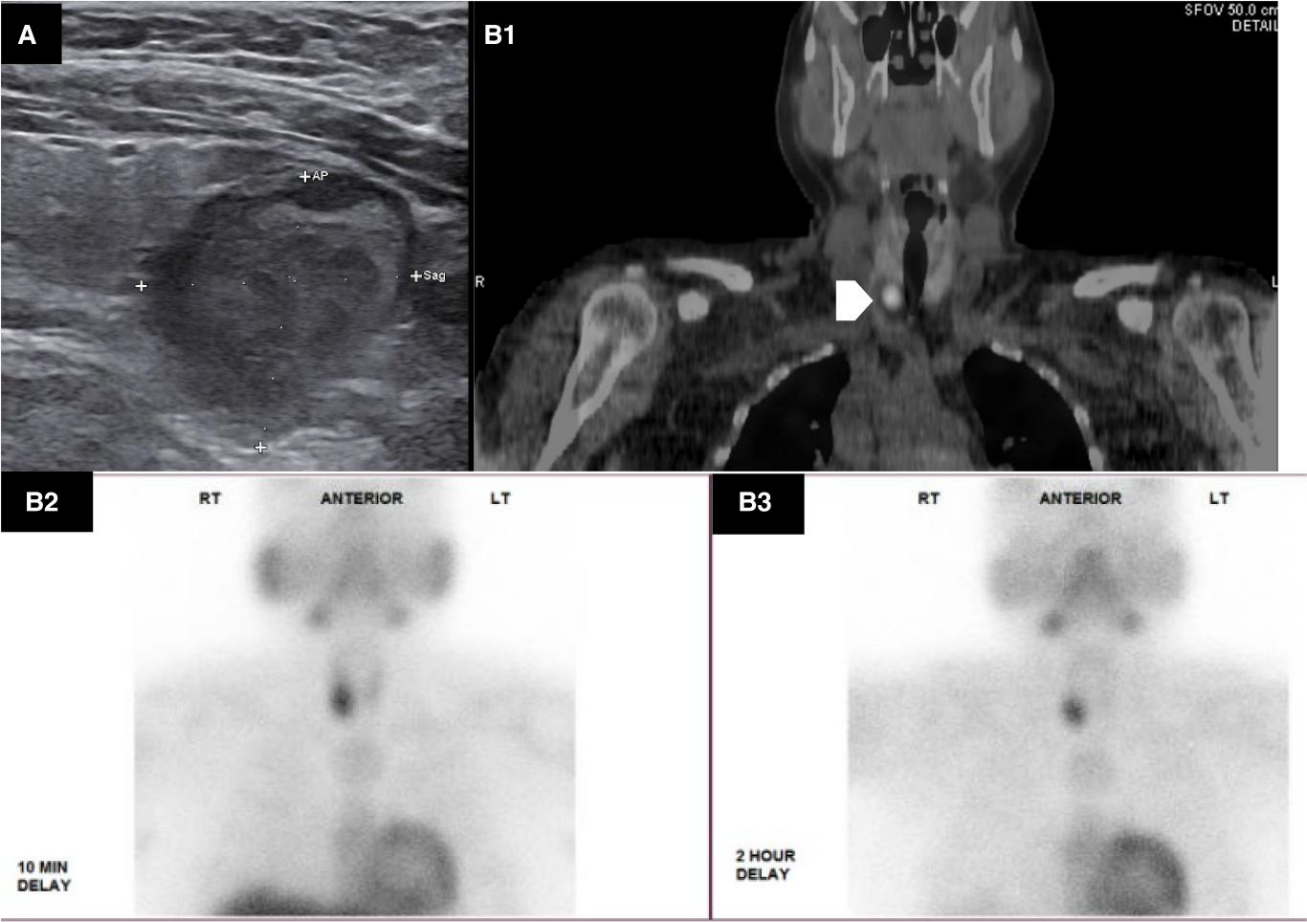
Preoperative imaging assessment. Preoperative ultrasound (A) showed a 1.95 × 1.25 × 5.31 cm right inferior parathyroid lesion. This lesion was also identified by sestamibi scan with 4-dimensional computed tomography (B1, white arrowhead) and showed increased radiotracer activity on 2-hour delayed planar imaging (B2-B3, where B2 represents 10-minute delay and B3 represents 2-hour delay).

Head and neck ultrasound showed enlarged left and right inferior parathyroid glands, measuring 1.52 cm and 2.69 cm, respectively (Fig. 1). A nuclear medicine parathyroid scan with computed tomography showed increased uptake in the right inferior thyroid that persisted on 2-hour delayed imaging, consistent with a parathyroid lesion (Fig. 1).

The patient underwent 4-gland exploration with noted gross enlargement of all 4 parathyroid glands. A 3.5-gland parathyroidectomy was performed, with intraoperative PTH dropping from 3035 pg/mL (SI: 321.8 pmol/L) (reference range, 14-72 pg/mL [SI: 1.5-7.6 pmol/L]) to 115 pg/mL (SI: 12.2 pmol/L) at 10 minutes postexcision, indicative of adequate surgical resection. A 30 mg parathyroid remnant was left in place, with nerve signaling and blood supply to the remnant gland confirmed intraoperatively and considered adequate. Surgical pathology confirmed a 9.5 g parathyroid adenoma of the right inferior gland and hypercellular tissue in the remaining glands. He was started on calcium carbonate and calcitriol on postoperative day (POD) 0 and developed hypocalcemia on POD 1.

## Diagnostic assessment

**Figure 2:**
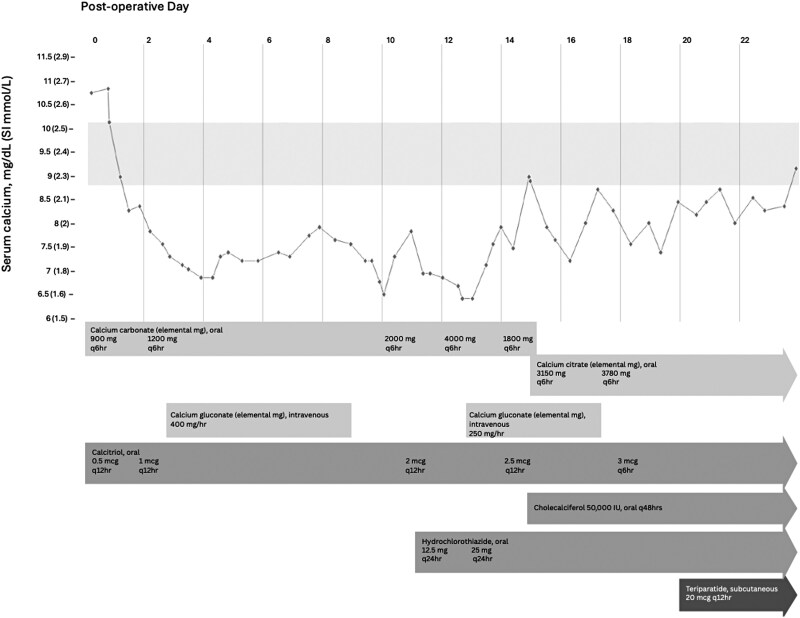
Postoperative calcium trends and medical interventions. Calcium, mg/dL (SI mmol/L) trends of the patient while hospitalized with each calcium or vitamin D-related treatment therapy initiation and duration. Normal serum calcium was defined as 8.7 to 10.2 mg/dL (SI: 2.17-2.54 mmol/L) by the institution's laboratory and denoted by the gray shaded box. Significant hypocalcemia was seen during the first 9 days, requiring continuous intravenous calcium gluconate infusion, with improvements in calcium levels occurring with the initiation of calcium citrate but sustained response only occurring with the addition of teriparatide. Calcium doses are expressed in mg of elemental calcium. Frequency of dosing is expressed by frequency of dosing per hours (hr), where q means “every.” Abbreviation: SI, International System of Units.

Over the course of 2 weeks, the patient continued to have hypocalcemia despite increases in oral calcium carbonate and calcitriol, and required continuous intravenous (IV) calcium infusion (Fig. 2). He was started on hydrochlorothiazide (HCTZ) POD 11 and switched to calcium citrate on POD 15. He was receiving calcium citrate 3780 mg every 6 hours (15.12 g of elemental calcium/day), calcitriol 3 mcg every 6 hours (12 mcg/day), cholecalciferol 50 000 international units 3 times a week, and HCTZ 25 mg daily but continued to have hypocalcemia with symptomatic paresthesias when calcium infusion was stopped. PTH was measured on POD 12 and was inappropriately normal at 44 pg/mL (SI: 4.7 pmol/L). Spot urine calcium on POD 17 was undetectable, and subsequent 24-hour urine calcium was also undetectable (urine volume 3.7 L). Serum phosphate remained in the normal range, but magnesium required supplementation. The combination of hypocalcemia with normal PTH despite high doses of calcium (>15 g/day), calcitriol, HCTZ, and cholecalciferol was concerning for HBS.

## Treatment

In the setting of hypocalcemia refractory to standard therapies and paresthesias only remediable with IV calcium, the inpatient endocrinologist requested approval for off-label use of teriparatide by the hospital pharmacy, with the rationale that continuous calcium infusion and long-term hospitalization carried more risks, costs, and burden for the patient and hospital. The patient was started on teriparatide 20 mcg every 12 hours on POD 20, with subsequent improvement in serum calcium levels between 8 and 9 mg/dL (SI: 2-2.25 mmol/L). He was discharged on POD 24 with electrolyte supplementation and teriparatide 20 mcg every 12 hours, with a plan to decrease teriparatide to daily after 1 week. The dosing strategy of teriparatide was based on a previously published study using the medication for postoperative hypocalcemia after thyroidectomy [6].

## Outcome and follow-up

The patient’s outpatient labs the week of discharge showed serum calcium at the goal of 8.0 to 8.5 mg/dL (SI: 2-2.12 mmol/L). He reported intermittent paresthesias that improved with taking a scheduled calcium supplement. About 2 months after surgery, teriparatide was no longer covered due to off-label use. Insurance approval was obtained for off-label use of palopegteriparatide 18 mg daily. Palopegteriparatide was uptitrated to 24 mg daily due to persistent mild hypocalcemia. He had subsequent improvement in calcium levels that allowed for decreased supplementation. His alkaline phosphatase improved to 120 U/L (SI: 2 μkat/L) 2 months postoperatively. Three months after starting palopegteriparatide, he was able to come off HCTZ, vitamin D and magnesium, and was maintained on elemental calcium 1 g 2 times per day and calcitriol 1 mcg 2 times per day, with serum calcium between 8.6 and 9.6 mg/dL (SI: 2.15-2.4 mmol/L).

## Discussion

In this case, we present a patient with hyperparathyroidism who underwent 3.5 gland parathyroidectomy with subsequent severe hypocalcemia and HBS lasting for more than 2 weeks despite aggressive use of standard therapies who had clinical and biochemical improvement with PTH analog initiation. This is 1 of only a few cases in the literature to successfully use PTH analogs in the management of HBS after parathyroidectomy.

PTH analogs are not considered first-line agents for acute postoperative hypocalcemia management largely due to cost and lack of US Food and Drug Administration (FDA) approval for this indication. In the past decade, there have been a few studies investigating early use of PTH analogs in the management of postoperative hypocalcemia following thyroidectomy that showed decreased hospitalization duration and improved clinical outcomes in those treated with PTH analogs [6-8]. However, these studies are notably different from the present case, as patients with preexisting parathyroid disease were excluded. This is important because those with hyperparathyroidism are likely at higher risk for postoperative hypocalcemia due to possible suppression of the remaining parathyroid glands and shifts in skeletal calcium homeostasis; however, further studies are needed to elucidate this.

Andrysiak-Mamos et al [8] reported a case of severe hypocalcemia and HBS following parathyroidectomy requiring >10 g elemental calcium per day with normal serum calcium levels only being achieved 72 hours after initiating teriparatide. In this case and ours, patients had prolonged hospitalizations of 11 and 3 weeks, respectively, were dependent on IV calcium infusion for symptomatic hypocalcemia, and rapidly achieved safety parameters for discharge after starting PTH analogs. Additionally, in both cases the burden of calcium supplementation decreased with initiation of teriparatide, and this may be of particular interest when considering long-term patient adherence to these pill-burdensome regimens.

There is insufficient evidence-based data to define a threshold to use PTH analogs. Based on this case and similar cases in the literature [7, 8], it may be reasonable to consider if elemental calcium supplementation is reaching 10 to 15 g/day, if frequent IV supplementation is needed to prevent tetany, if early postoperative PTH levels are <10 pg/mL (SI: 1.1 pmol/L) [7], or if there is >95% decrease in PTH postoperatively; however, further investigations are required to validate these observations.

There is evidence-based data to predict HBS based on preoperative clinical features including low vitamin D levels, elevated alkaline phosphatase, and bony pain [4]. Studies have demonstrated the benefits of pretreatment with bisphosphonates [9-11] and of vitamin D replacement [12]. Additionally, alternative surgical interventions can be considered. One study found that limited dissection of 1 or 2 parathyroid glands compared to subtotal or total parathyroidectomy resulted in less postoperative hypocalcemia and similar rates of recurrent hypercalcemia [13]. Another approach may be to seek approval preoperatively for PTH analogs in high-risk cases.

Cost remains a major barrier to the use of PTH analogs, both inpatient and outpatient. However, we would argue that the cost of prolonged hospitalization would likely outweigh the cost of acute PTH analog use. The cost of ongoing teriparatide use is harder to elucidate, as the duration of hypocalcemia and HBS appears variable between cases [8]. Difficulty obtaining outpatient coverage may be a barrier for treatment for those with ongoing hypocalcemia, especially for those who are not yet diagnosed with chronic hypoparathyroidism, defined as hypoparathyroidism lasting for more than 6 months [1]. There are currently no PTH analogs with FDA approval for management of acute postoperative hypocalcemia [14-16], and only palopegteriparatide is approved for chronic hypoparathyroidism. The lack of a FDA indication for acute postoperative hypocalcemia can significantly impact coverage and practical use of these medications. It is likely that coverage would be variable based on severity of disease and factors related to the strength of prior authorizations submitted by physicians, which may not result in equitable disease treatment. Further studies are needed to study the use of these therapies in the acute setting, and may result in changes in indications and coverage in the future.

In conclusion, we present a patient with hyperparathyroidism who developed HBS refractory to high doses of calcium, calcitriol, and vitamin D supplementation than previously described. He only became clinically stable for discharge once teriparatide was initiated. We highlight the clinical impact of teriparatide and palopegteriparatide in the treatment of acute postoperative hypocalcemia and add to a small body of work in the literature documenting a successful response in this severe disease to early PTH analog initiation.

## Learning points

- PTH analogs can be successfully used to manage severe, acute postoperative hypocalcemia mediated by HBS.
- Lack of FDA approval for PTH analogs for acute hypocalcemia management may limit coverage for these therapies.
- Further research investigating these agents for acute hypocalcemia is warranted and may result in improved coverage.

## Contributors

All authors made individual contributions to authorship. N.S. was involved with the diagnosis and management of the patient and critical review of the manuscript. S.J. was involved with conception and design, data acquisition and drafted the manuscript. J.R. was involved with conception and design, and critical review of the manuscript. All authors reviewed and approved the final draft.

## Data Availability

Data sharing is not applicable to this article as no datasets were generated or analyzed during the current study.
